# Carotenoids Composition of Green Algae *Caulerpa racemosa* and Their Antidiabetic, Anti-Obesity, Antioxidant, and Anti-Inflammatory Properties

**DOI:** 10.3390/molecules28073267

**Published:** 2023-04-06

**Authors:** Rudy Kurniawan, Fahrul Nurkolis, Nurpudji Astuti Taslim, Dionysius Subali, Reggie Surya, William Ben Gunawan, Darmawan Alisaputra, Nelly Mayulu, Netty Salindeho, Bonglee Kim

**Affiliations:** 1 Alumnus of Internal Medicine, Faculty of Medicine, University of Indonesia—Cipto Mangunkusumo Hospital, Jakarta 10430, Indonesia; rudycrates@gmail.com; 2 Department of Biological Sciences, State Islamic University of Sunan Kalijaga (UIN Sunan Kalijaga), Yogyakarta 55281, Indonesia; fahrul.nurkolis.mail@gmail.com; 3 Division of Clinical Nutrition, Department of Nutrition, Faculty of Medicine, Hasanuddin University, Makassar 90245, Indonesia; 4 Department of Biotechnology, Faculty of Biotechnology, Atma Jaya Catholic University of Indonesia, Jakarta 12930, Indonesia; dionysius.subali@atmajaya.ac.id; 5 Department of Food Technology, Faculty of Engineering, Bina Nusantara University, Jakarta 11480, Indonesia; reggie.surya@binus.edu; 6 Alumnus of Nutrition Science, Faculty of Medicine, Diponegoro University, Semarang 50275, Indonesia; wbwilliambenwb@gmail.com; 7 Department of Chemistry, State Islamic University of Sunan Kalijaga (UIN Sunan Kalijaga), Yogyakarta 55281, Indonesia; 18106030024@student.uin-suka.ac.id; 8 Department of Nutrition, Universitas Muhammadiyah Manado, Manado 95249, Indonesia; nmayulu@unsrat.ac.id; 9 Fishery Products Technology Study Program, Faculty of Fisheries and Marine Sciences, Sam Ratulangi University, Manado 95115, Indonesia; nettysalindeho0312@unsrat.ac.id; 10 Department of Pathology, College of Korean Medicine, Kyung Hee University, Hoegidong Dongdaemun-gu, Seoul 02447, Republic of Korea; bongleekim@khu.ac.kr; 11 Korean Medicine-Based Drug Repositioning Cancer Research Center, College of Korean Medicine, Kyung Hee University, Seoul 02447, Republic of Korea

**Keywords:** green algae carotenoid, *Caulerpa*, antidiabetic, anti-obesity, anti-oxidative, anti-inflammatory, cytotoxicity properties, functional food, nutraceuticals

## Abstract

Green alga *Caulerpa racemosa* is an underexploited species of macroalgae, even though it is characterized by a green color that indicates an abundance of bioactive pigments, such as chlorophyll and possibly xanthophyll. Unlike chlorophyll, which has been well explored, the composition of the carotenoids of *C. racemosa* and its biological activities have not been reported. Therefore, this study aims to look at the carotenoid profile and composition of *C. racemose* and determine their biological activities, which include antidiabetic, anti-obesity, anti-oxidative, anti-inflammatory, and cytotoxicity in vitro. The detected carotenoids were all xanthophylls, which included fucoxanthin, lutein, astaxanthin, canthaxanthin, zeaxanthin, β-carotene, and β-cryptoxanthin based on orbitrap-mass spectrometry (MS) and a rapid ultra-high performance liquid chromatography (UHPLC) diode array detector. Of the seven carotenoids observed, it should be highlighted that β-carotene and canthaxanthin were the two most dominant carotenoids present in *C. racemosa*. Interestingly, the carotenoid extract of *C. racemosa* has good biological activity in inhibiting α-glucosidase, α-amylase, DPPH and ABTS, and the TNF-α and mTOR, as well as upregulating the AMPK, which makes it a drug candidate or functional antidiabetic food, a very promising anti-obesity and anti-inflammatory. More interestingly, the cytotoxicity value of the carotenoid extract of *C. racemosa* shows a level of safety in normal cells, which makes it a potential for the further development of nutraceuticals and pharmaceuticals.

## 1. Introduction

Around 537 million people worldwide live with diabetes. This number is predicted to rise to 783 million by 2045 [1,2,3]. Obesity, which is part of the metabolic syndrome along with diabetes, also needs attention. More than 1 billion people worldwide are obese, and the number is still increasing. Obesity leads to a range of cardiometabolic syndromes, including diabetes [4]. The incidence of obesity and diabetes are interrelated, resulting from a constellation of metabolic dysfunctions, including glucose intolerance, dyslipidemia, oxidative stress and inflammation, hypertension, insulin resistance, and intra-abdominal adiposity [5,6].

Various antidiabetic and metabolic syndrome drug innovations are continuously being developed to reduce the number of diabetes and related diseases. Metabolic syndrome treatment is quite complex, considering the aspects of cost and availability. Unfortunately, the treatment, especially the medication, is not evenly distributed and is relatively unaffordable [7]. Countries with a higher per capita income tend to receive therapy more easily. However, the fact is that over three in four adults with diabetes live in low- and middle-income countries [8]. Therapeutic innovations based on natural product ingredients can offer an alternative approach that is relatively inexpensive and easily accessible in managing metabolic syndrome.

One of the growing therapeutic innovations is the functional food industry. This trend is growing with increasing research, publication, and public awareness of the close relationship between food, nutrition, and health. Indonesia, as a maritime country, has more than 30,000 species of plants and marine resources, but only a few have been investigated and utilized optimally [9]. In fact, we are faced with a condition where many Indonesian people still think that medicines derived from nature are more acceptable [10]. Unfortunately, currently, there are still many herbal treatments that are not standardized. One that caught our attention was the Indonesian green algae *Caulerpa racemosa* (green seaweed or sea grapes).

*C. racemosa* has been widely studied for its role in lowering blood sugar and improving the lipid profile, anti-inflammatory, and antioxidants [11,12,13]. According to a previous study, caulerpin, an indole alkaloid of *C. racemosa*, may sensitize insulin, but a clear mechanism has not been established [14,15,16,17]. It needs to be further explored through scientific research to provide new insights and synergistic effects in the utilization of *C. racemosa* as a functional food candidate.

*C. racemosa* contains not only vitamins, minerals, protein, lipids, and polysaccharides but also various polyphenols, antioxidants, and pigments. The potential antidiabetic effects could partly be contributed to the range of antioxidant compounds found in seaweeds and algae. One of these is carotenoid, such as fucoxanthin (a marine carotenoid), which can be found abundantly in seaweeds and microalgae [18,19]. Carotenoids are one of the essential antioxidant agents, which have the characteristics of a linear C40 chain, containing up to eleven conjugated bonds (allenic bonds) that can participate in an antioxidant activity through the transfer of excess singlet oxygen energy in the central allenic long chain [20,21]. Interestingly, green algae contain a lot of chlorophyll pigment, which contributes to giving it its color [22]. It is suspected that carotenoids, which belong to the xanthophyll group, are also abundant in this green algae. However, until now, studies that have successfully reported the composition of carotenoids and their derivatives in green algae are still few, and further exploratory studies need to be carried out. Therefore, this study was conducted to evaluate the carotenoid composition of green alga *C. racemosa* and its potential in vitro effect on diabetes and obesity, its radical scavenging activities, and its anti-inflammatory properties. In addition, the potential safety value for consumption through in vitro cytotoxicity in normal human cells is also reported in this work.

## 2. Results

### 2.1. Quantitation and Identification of Caulerpa racemosa Carotenoids Content

Table 1 represents the presence of the targeted carotenoids in *C. racemosa* by orbitrap-mass spectrometry (MS), and their identity was confirmed by authentic standard compounds. The detected carotenoids were all xanthophylls, which included fucoxanthin, lutein, astaxanthin, canthaxanthin, zeaxanthin, and β-cryptoxanthin. In this study, the MS system applied with ESI negative–positive mode ionized the xanthophylls well but not the hydrocarbon carotenes (such as β-carotene and lycopene). Hence, the rapid ultra-high performance liquid chromatography (UHPLC) diode array detector was utilized to detect and quantify the presence of β-carotene in *C. racemosa*.

The quantification of the present carotenoids observed in *C. racemosa* is shown in Table 2. Three different solvents were used to extract the carotenoids from the *C. racemosa*: ethanol (EtOH) to extract the polar compounds (CrE), ethyl acetate to extract the semi-polar compounds (CrEA), and *n*-hexane to extract the non-polar compounds (CrNH). It is noteworthy that each carotenoid may have a different profile of solubility. Cantaxanthin and β-carotene are the most dominant carotenoids in the polar fraction (15.70 and 20.50 mg/100 g, respectively). They are present in relatively low amounts in semi-polar and non-polar solvents. Interestingly, other carotenoids, including β-cryptoxanthin, fucoxanthin, astaxanthin, zeaxanthin, and lutein, were the most extracted in a non-polar solvent and very little present in the polar fraction. Among the carotenoids present in the non-polar fraction, zeaxanthin appeared as the most abundant (9.00 mg/100 g). Overall, β-carotene and canthaxanthin were the two most dominant carotenoids present in the *C. racemosa.*

All carotenoids were quantified based on their retention times compared with authentic standards using rapid ultra-high performance liquid chromatography-electrospray ionization tandem mass spectrometry (UHPLC-ESI-MS). Furthermore, all carotenoids observed in *C. racemosa* are visualized in Table S1.

### 2.2. In Vitro Antidiabetic Evaluation via Carbohydrate (α-Glucosidase, α-Amylase) Hydrolyzing Enzyme Inhibition

**Figure 1:**
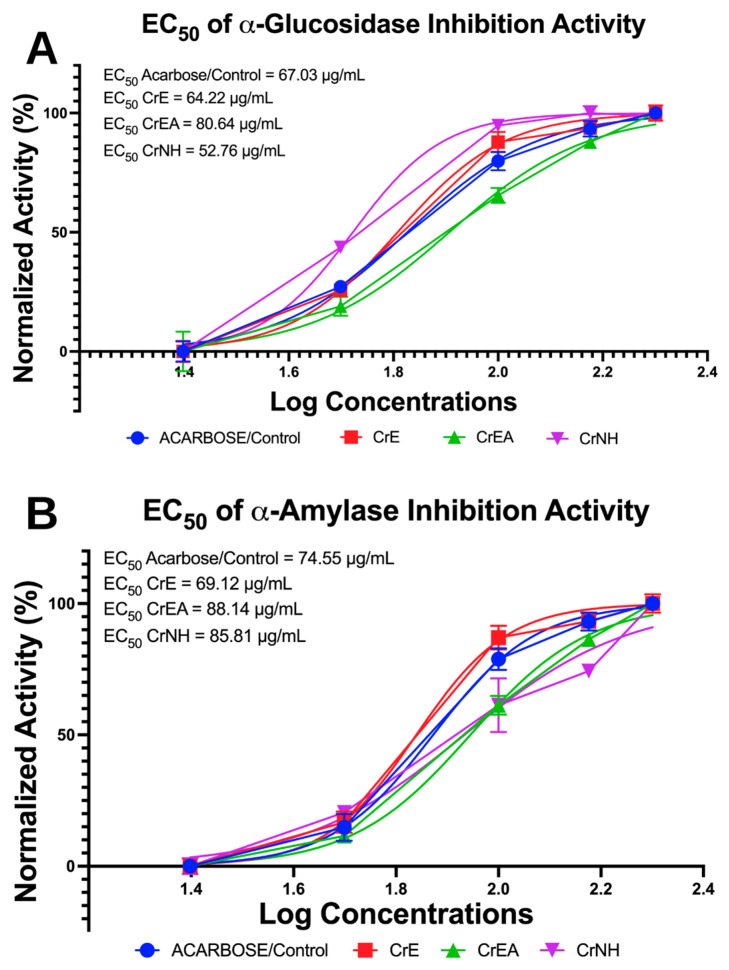
Antidiabetic potential of the carotenoid extract of *C. racemosa* (CEC) via in vitro enzymatic assay. (**A**). The half-maximal effective concentration (EC_50_) of α-glucosidase inhibition. (**B**). The EC_50_ of α-amylase inhibition. CrE: *C. racemosa*—ethanol (polar); CrEA: *C. racemosa*—ethyl acetate (semi-polar); CrNH: *C. racemosa*—*n*-hexane (non-polar).

As a carbohydrate hydrolyzing enzyme, the inhibition of both α-glucosidase and α-amylase enzymes is an approach to determining antidiabetic activity in vitro. The inhibition activity of α-glucosidase and α-amylase was measured to calculate the EC_50_ value (Figure 1). These values indicate the potential concentration of *C. racemosa* carotenoid extract using polar (CrE), semi-polar (CrEA), or non-polar (CrNH) solvents as an antidiabetic candidate compared with acarbose (the standard drug or control). The CrNH showed the highest efficacy for inhibiting the α-glucosidase activity, with an EC_50_ value of 52.76 µg/mL (Figure 1A). For the α-amylase inhibition, the CrE showed the highest efficacy, with an EC_50_ value of 69.12 µg/mL (Figure 1B).

### 2.3. In Vitro Anti-Obesity Evaluation via Lipid (Lipase) Hydrolyzing Enzyme Inhibition

**Figure 2:**
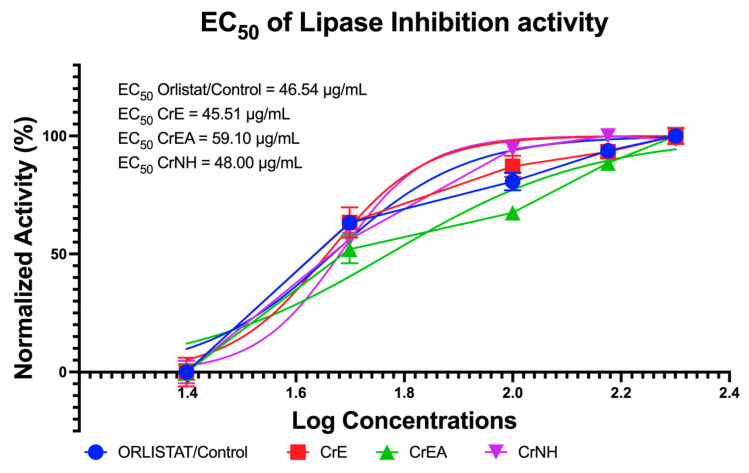
The lipase inhibition activity of carotenoid extract of *C. racemosa*. CrE: *C. racemosa*—ethanol (polar); CrEA: *C. racemosa*—ethyl acetate (semi-polar); CrNH: *C. racemosa*—*n*-hexane (non-polar).

Lipase inhibitors have received great attention from researchers as an anti-obesity determination in recent years. Moreover, lipase inhibitors from natural ingredients have become of great interest because of their structural diversity and relatively low toxicity. The inhibition activity of lipase was measured to calculate the EC_50_ value. These values indicate the potential concentration of *C. racemosa* carotenoid extract using polar, semi-polar, or non-polar solvents as an anti-obesity candidate compared with orlistat. The carotenoid extract of *C. racemosa* in a polar solvent (CrE) showed the highest efficacy for inhibiting lipase activity, with an EC_50_ value of 45.51 µg/mL (Figure 2).

### 2.4. In Vitro Cytotoxicity Evaluation via MTT Assay in Normal Cell Lines

The effectiveness as a candidate for nutraceuticals and/or pharmaceutical agents needs to be addressed for its safety in terms of cytotoxicity in normal cells. In addition, apart from having antidiabetic and anti-obesity activity, the carotenoid extract of *C. racemosa* is safe for further consumption based on cytotoxicity tests on BUD-8 normal cell lines, which are summarized in Table 3. All the values are the half-maximal lethal concentration (LC_50_) value of the carotenoid extract of *C. racemosa* on a cytotoxicity test in the BUD-8 normal cell lines (>500 μg/mL) (Table 3). 

### 2.5. Antioxidant Capabilities Evaluation via DPPH and ABTS Inhibition Assays

**Figure 3:**
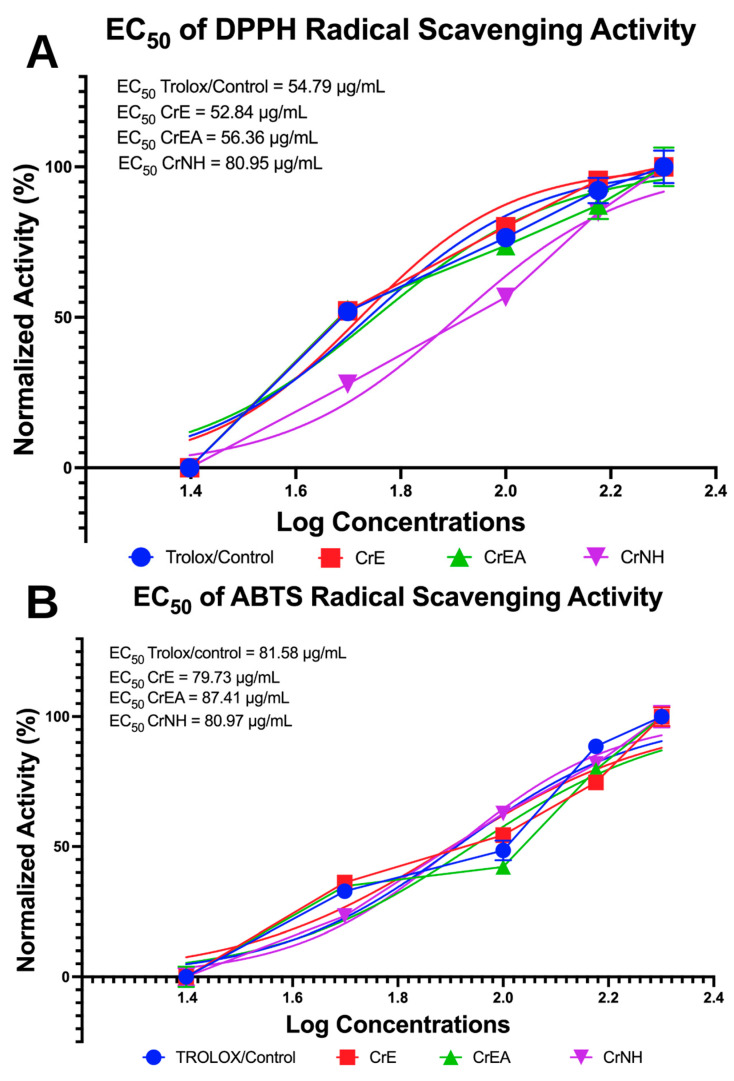
Antioxidants activity of carotenoid extract of *C. racemosa*. (**A**). The half-maximal effective concentration (EC_50_) of DPPH inhibition. (**B**). The EC_50_ of ABTS inhibition. CrE: *C. racemosa*—ethanol (polar); CrEA: *C. racemosa*—ethyl acetate (semi-polar); CrNH: *C. racemosa*—*n*-hexane (non-polar).

The inhibition activity of DPPH and ABTS as radical was measured to calculate the EC_50_ value and determines the antioxidant capabilities of the carotenoid extract of *C. racemosa* (shown in Figure 3). In the DPPH radical scavenging activity assay, it shows that the carotenoid extract of the polar solvent fraction *C. racemosa* (CrE) showed the highest efficacy, with an EC_50_ value of 52.84 µg/mL, which is more potent compared to Trolox as a control (Figure 3A). More interestingly, the CrE and carotenoid extract of *C. racemosa* in the non-polar solvent/*n*-hexane fraction showed high efficacy activity, even when compared to the standard antioxidant/control (Trolox) (Figure 3B), with an EC_50_ value of 79.73 µg/ mL and 80.95 µg/ mL, respectively.

### 2.6. Anti-Inflammatory Evaluation of Carotenoid Extract of C. racemosa

**Figure 4:**
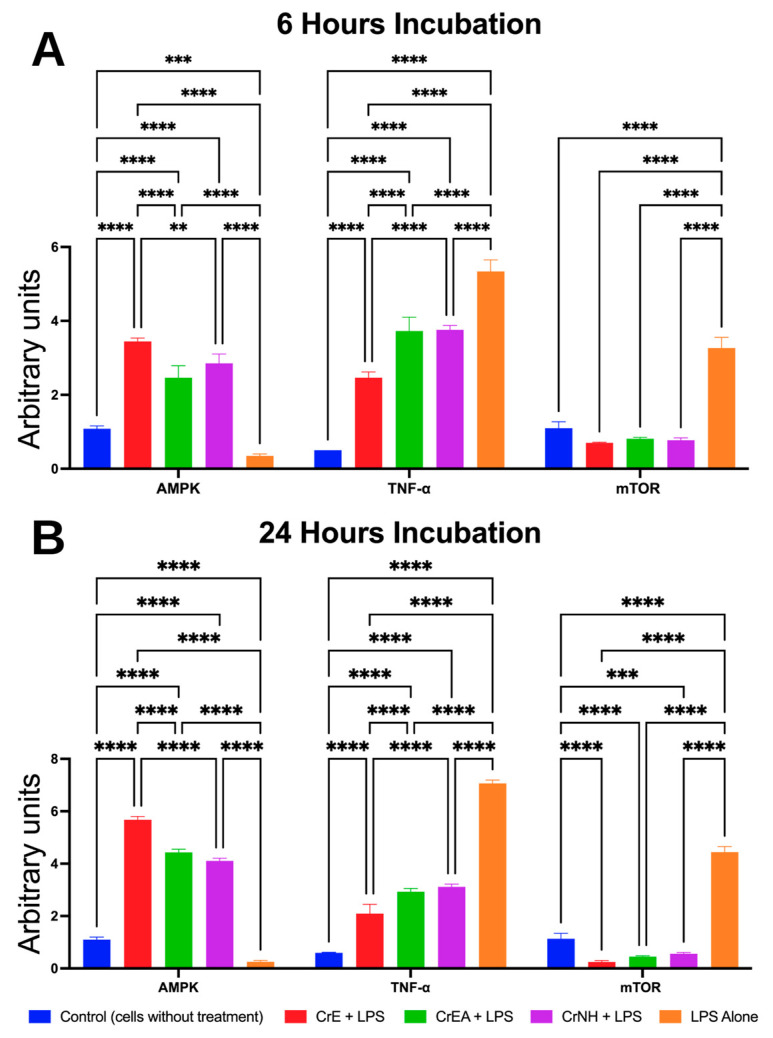
Expression of the AMP-activated protein kinase (AMPK), tumor necrosis factor-alpha (TNF-α), and mammalian target of Rapamycin (mTOR) kinase regulated by carotenoid extract of *C. racemosa*. (**A**) Expression of inflammatory markers at 6 h of incubation. (**B**) Expression of inflammatory markers at 24 h of incubation. CrE: *C. racemosa*—ethanol (polar); CrEA: *C. racemosa*—ethyl acetate (semi-polar); CrNH: *C. racemosa*—*n*-hexane (non-polar); control lipopolysaccharide (LPS). ** *p* = 0.0065; *** *p* = 0.003; **** *p* < 0.0001.

Figure 4 presents the data on the modulation of inflammatory biomarkers by the three carotenoid extracts of *C. racemosa*. Consistent with theory, lipopolysaccharide (LPS) induction in RAW264.7 cells significantly upregulated the TNF-α and mTOR and was accompanied by the downregulation of the AMPK expression at both 6 and 24 h of incubation (Figure 4). At six hours of incubation, the expression of the AMPK was significantly elevated compared to the control cells without treatment or the control-LPS alone (*p* < 0.0001) (Figure 4A). The CrE demonstrated a stronger effect on the increase of the AMPK expression compared to the CrEA and CrNH (*p* = 0.0065; *p* < 0.0001). In line with the result of the AMPK, the CrE showed a greater impact on downregulating the expressions of the TNF-α and mTOR compared to the CrEA and CrNH (*p* < 0.0001). In general, the CrE, CrEA, and CrNH were also observed to downregulate the TNF-α and mTOR expressions at 6 h of incubation in the LPS-induced cells (Figure 4A). 

The analysis of the inflammatory biomarker expressions at 24 h revealed that the expression of the AMPK was significantly increased in all the experimental groups compared to the control group (*p* < 0.0001) (Figure 4B). Consistent with the results at 6 h of incubation, the CrE demonstrated a significantly stronger effect on the increase of the AMPK expression compared to the other groups (*p* < 0.0001). Interestingly, all of the treatments by the carotenoid extract of *C. racemosa* were able to reduce the TNF-α and mTOR expressions significantly in LPS-induced cells, even though the CrE still led to its most effective downregulating activity (Figure 4B).

The summary of changes in the inflammatory biomarker expression is summarized in Table 4. In general, the carotenoid extract of *C. racemosa* increased the expression of the AMPK and decreased the expression of the TNF-α and mTOR. The CrE caused a higher elevation of the AMPK expression in comparison to the other groups’ treatment within either six or twenty-four hours of incubation.

## 3. Discussion

Marine algae (or seaweed) are commonly consumed in Asian countries, such as Korea and Indonesia. One of the underexploited types of marine algae is *Caulerpa racemosa*, more specifically known as green algae. Despite previous studies reporting the efficacies of the extract of *C. racemosa,* the profiling and biological activity of carotenoids present in the extract of *C. racemosa* have not been clearly elucidated. As previously hypothesized, we suspected that *C. racemosa* contained carotenoids and their derivatives. Carotenoids are categorized into two classes, xanthophylls with oxygen in their molecular structures and carotenes with hydrocarbon structures [23]. In this current study, we reported seven types of xanthophylls in *C. racemosa* and its fractions, including fucoxanthin, lutein, astaxanthin, canthaxanthin, zeaxanthin, β-carotene, and β-cryptoxanthin. Among the detected xanthophylls, β-carotene and canthaxanthin appeared as the most abundant carotenoids. The quantity of elucidated carotenoids was dependent on the polarity of the solvents used in the extraction process, and this is in line with another study [24]. Fucoxanthin, which is also the dominant carotenoid in brown algae, has been demonstrated to support medical treatments against several diseases, including metabolic syndrome, diabetes, and obesity-related diseases [25,26,27]. Several systematic studies have reported that zeaxanthin, lutein, and meso-zeaxanthin possess protective effects in age-related macular degeneration and adjunctive nutraceutical strategy [28].

Obesity has been a risk factor for global public health in relation to degenerative diseases, such as diabetes, hypertension, and cardiometabolic diseases [29,30,31]. In the present study, we showed the antidiabetic and anti-obesity effects of *C. racemosa* in vitro. In previous studies, fucoxanthin was reported to induce the expression of the uncoupling protein 1 (a protein of the inner membrane of the mitochondria), as well as to stimulate energy expenditure by promoting fatty acid oxidation and heat generation [32,33]. These findings were aligned with the antioxidant activity of *C. racemosa,* as confirmed by the DPPH and ABTS assays. Another study by Nishikawa et al. clarified the role of fucoxanthin in improving insulin resistance and normalizing blood glucose levels through the regulation of adipocytokine in white adipose tissues and glucose transporter-4 in skeletal muscle cells [34]. In the present study, we highlighted the antidiabetic activity of carotenoids extracted from *C. racemosa* by observing their inhibitory effects against α-amylase and α-glucosidase. Some carotenoids have antioxidant activity due to the presence of long conjugated double bonds that can delocalize unpaired electrons, thus preventing them from being unstable free radicals [35,36,37]. Here, we have confirmed that carotenoids extracted from *C. racemosa* could be a novel compound with a health-related potential to prevent metabolic syndrome, in addition to carotenoids from brown algae.

Obesity, diabetes, and metabolic syndromes may disturb the body’s biomechanism and promote inflammation [38,39]. The hypermetabolic activity could lead to oxidative stress, organelle hypertrophy, and inflammation. A meta-analytic study showed that oxidative stress and the hyperconsumption of fat and/or other macronutrients without antioxidant supplementation favored inflammation related to obesity and diabetes [40]. To overcome such situations, the consumption of antioxidants appears primordial to compensate for oxidative stress and reduce inflammation. Natural products can be an option for antioxidant supplements. The present study demonstrated the potential of the carotenoids from *C. racemosa* as a natural inflammation suppressor by modulating the AMPK-mTOR-TNF-α signaling pathway. In addition, clinical activation of the AMPK has been proven to relieve inflammation-related pain by inhibiting the activation of NF-κB, mTOR, and IL-1β [41]. Lastly, the improved immunomodulatory effect can also be obtained through the consumption of functional food [42]. 

Since this is a preliminary study that has reported on the health-related potential of the carotenoid ingredients extracted from Indonesian green algae *C. racemosa,* further studies are needed to isolate and purify each of the observed carotenoids. The limitation of the present study is related to the in silico or molecular simulation study since the molecular structure of each observed carotenoid would need further programming for molecular docking.

## 4. Materials and Methods

### 4.1. Preparation of Green Algae Caulerpa racemosa

Green algae (*Caulerpa racemosa*) were collected from a cultivation pond in Jepara Regency, Central Java Province, Indonesia, with the permission of the pond owner and local authorities. The botanical identification and authentication were confirmed at the State Islamic University of Sunan Kalijaga Yogyakarta (Universitas Islam Negeri Sunan Kalijaga), Yogyakarta, Indonesia, using the National Center for Biotechnology Information Taxonomy ID (*C. racemosa* = 6968) and Integrated Taxonomic Information System-Report ID (*C. racemosa* = 6968). The green algae were then thoroughly washed to remove any dirt or debris and then sun-dried for two to three days before being dried in an oven at 40 °C. The dried green algae were then chopped into small pieces and pulverized in a blender to produce green algae Simplica powder. The protocol in this section refers to previous studies [13,43], and maceration was used to extract the powder for further research.

### 4.2. Extraction of Green Algae Caulerpa racemosa

**Figure 5:**
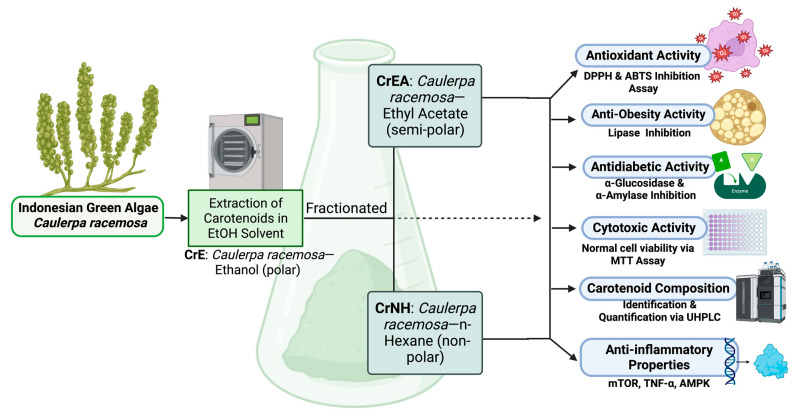
Methodical schematic of carotenoid extract *Caulerpa racemosa* study flow.

To prepare the green algae (*C. racemosa*) carotenoids extract powder for research and analysis, one kilogram of the Simplica powder from each green algae was mixed with 2 L of 96% ethanol solvent in a ratio of 1:2 and placed in a dark bottle. The Simplica was soaked for three periods of 24 h, with the obtained filtrate stirred and filtered every 24 h using Whatman 41 paper. The residue was then soaked in a new 96% ethanol (EtOH) solvent, and the process was repeated and followed by sonicating for 30 min (40 °C) using an ultrasound sonicator (400 W, Branson 2510 model; Danbury, CT, USA). The resulting extract was concentrated using a rotary evaporator under low pressure (100 millibars) for 90 min and evaporated in a 40 °C oven, and sequentially partitioned into equal volumes using ethyl acetate (EtOAc) and *n*-hexane solvents to produce a viscous extract that included CrE: *Caulerpa racemosa*—ethanol (polar); CrEA: *Caulerpa racemosa*—ethyl acetate (semi-polar); CrNH: *Caulerpa racemosa*—*n*-hexane (non-polar), referring to previous studies [13,43]. The flow of this study is presented in Figure 5.

### 4.3. Carotenoid Identification and Analysis of Caulerpa racemosa via UHPLC-ESI-MS/MS

First, the standard stock solution preparation protocol refers to those previously well-established [23]. We prepared a standard stock solution of 10 mg/mL individually by dissolving the analyte in a DCM/MeOH solution (50:50). The solution was stored at −20 °C in the dark. Standard stock solutions were used for spiking due to that different levels of spiking were required to validate the method. Only primary grade standards with a purity level of ~99.5% were used for further LC-MS/MS analysis. The chemical standards used included β-carotene, lutein, lycopene, β-cryptoxanthin, and zeaxanthin, which were purchased, obtained, and used according to the manufacturer, Sigma-Aldrich^®^ (St. Louis, MO, USA). In LC-MS analysis, *C. racemosa* extract was diluted with the DCM/MeOH solution (50:50) and filtered using a 0.22 μm nylon filter into LC vials to remove impurities before being injected into UHPLC at a concentration of 1 mg/mL. 

Second, the identification of carotenoids was carried out according to a modified protocol described in a previous study [23], using the UHPLC-ESI/HRMS/MSn system consisting of the Dionex UHPLC system Ultimate 3000 (Thermo Fisher Scientific, Waltham, MA, USA; Markham, Canada) and Q Exactive Hybrid Quadru-pole-Orbitrap Mass Spectrometer (Thermo Scientific, Waltham, MA, USA; Markham, Canada). MS-compatible C18 reversed-phase column (50 mm × 2.1 mm; at 1.7 μm particle size), Acquity UPLC BEH C18. The autosampler was set at 4 °C, with a sample volume of 3 μL loaded onto the column, which was run at 35 °C. The gradient program consisted of two mobile phases, water with 0.1% formic acid (A) and ACN with 0.1% formic acid (B). The flow rate was 0.3 mL/min, and the gradient program was 0–2 min, 5% B; 2–20 min, from 5% B to 99% B, 20–25 min, 99% B; 25–30 min 5% B. MS analysis was carried out in negative and positive ion electrospray ionization (ESI) mode. The mass spectrometer was used with a resolving power setting of 140,000 FWHM and a scanning range of 100–1000 m/z; the capillary temperature was set at 320 °C, and the envelope gas and auxiliary gas flow rates were 35 and 12 random units. The spray voltage was at 3.7 kV. The S-tube lens was regulated at 55 V for positive and negative ionization modes. MS/MS spectra were obtained using an impact energy of 35 V, and data were generated using X-Calibur 2.1.0 (Thermo Scientific, Waltham, MA, USA; Markham, Canada). Details of peak identification and UHPLC-MS/MS method validation in detail refer to the previous well-established publication [23].

### 4.4. In Vitro Antidiabetic Assay via α-Glucosidase and α-Amylase Inhibition (%)

The inhibitory activity was performed according to the previous literature [13,43]. The enzyme (76 UI, 1 mg) was mixed with a phosphate buffer (50 mL with pH 6.9) to obtain a concentration of 1.52 UI/mL. In the reaction tube, 0.35 mL of sucrose (65 mM), a maltose solution (65 mM), and samples (CrE, CrEA, and CrNH; quantities of 0.1 mL, 50 μg/mL, 100 μg/mL, 150 μg/mL, 200 μg/mL, and 250 μg/mL) were added, one at a time. After homogenization, α-glucosidase solution (1.52 UI/mL, 0.2 mL) was added to each tube, which was then maintained at thirty-seven degrees Celsius (37 °C) for 20 min. The enzyme was then inactivated and heated in a water bath for 2 min at 100 °C. Acarbose served as the positive control. To develop the color, 0.2 mL of a testing solution and a color reagent (3 mL) were used consecutively. Next, the system was warmed up to thirty-seven degrees Celsius (37 °C) for 5 min, and the solution absorption was examined at 505 nm afterward. The amount of glucose released during the reaction served as a marker of inhibitory activity.

The α-amylase (alpha-amylase) inhibition activity of the CrE, CrEA, and CrNH samples was measured based on the previous literature [15,44]. Diluted samples were incubated at five different concentrations (50 μg/mL, 100 μg/mL, 150 μg/mL, 200 μg/mL, and 250 μg/mL) for 10 min at 25 °C with a sodium phosphate buffer (0.02 M, pH 6.9) and 0.006 M of NaCl, as well as 0.5 mg/mL of a porcine pancreatic amylase. Then, 500 μL of a 1% starch solution in an assay buffer was added to each mixture. After 10 min of incubation at 25 °C, 3,5-dinitro salicylic acid was added to complete the process and incubated in a water bath at one hundred degrees Celcius (100 °C) for 5 min; the test tube was allowed to cool to 22 °C. To achieve values in the permissible range for recording the absorbance at 540 nm, dilution with distilled water (10 mL) was performed. The positive control used was acarbose.

### 4.5. In Vitro Anti-Obesity Evaluation via Lipase Inhibition Assay (%)

Crude pig pancreatic lipase (PPL, 1 mg/mL) was first dissolved in a 50 mM phosphate buffer (pH 7) before being centrifuged at 12,000× *g* to remove insoluble components. The creation of an enzyme stock (0.1 mg/mL) required a 10-fold dilution of the supernatant with a buffer. The lipase inhibition potential was assessed based on prior research. A transparent 96-well microplate containing 100 µL of CrE, CrEA, and CrNH samples was combined with 20 µL of 10 mM p-nitrophenyl butyrate (pNPB) in a buffer and incubated for 10 min at thirty-seven degrees Celsius (37 °C). The outcome was compared to the reference drug, orlistat (C_29_H_53_NO_5_, PubChem CID: 3034010), a well-known PPL or lipase inhibitor. Measurements were taken at 405 nm using a DR-200Bc ELISA microplate reader. The unit of activity was calculated using the yield of the reaction rate of 1 mol of p-nitrophenol (4-nitrophenol, C_6_H_5_NO_3_) per minute at thirty-seven degrees Celsius (37 °C). To measure the lipase inhibition activity, PPL activity was reduced in the test mixture by a specific amount. To ensure the validity of the study results, each sample was verified in thrice or triplicate (*n* = 3). The inhibitory data were obtained using the equation described previously by Permatasari et al. [13,43]:

(1) $$Inhibition\;of\;Lipase\;Activity\left(\%\right)=100-\frac{B-Bc}{A-Ac}\times 100\%$$

*A* = Activity without inhibitor; *B* = Activity with inhibitor; *Ac* = Negative control (−) without inhibitor; *Bc* = Negative control (−) with inhibitor.

### 4.6. Cytotoxicity Evaluation via MTT Assay 

A cytotoxicity test was carried out using a 3-(4,5-dimethylthiazol-2-yl)-2,5-diphenyl tetrazolium bromide (MTT) assay on a Caucasian human skin cell line (fibroblast normal cell; Bud-8) by assessing the viability of cells. After 24 h and 48 h of incubation, 100 µL of samples (CrE, CrEA, and CrNH) containing 100, 200, 300, 400, and 500 µg/mL was added to 100 µL of cells and dimethyl sulfoxide (DMSO; Sigma, Darmstadt, Germany) as the control, and complete details of this cytotoxicity test protocol refer to the previous well-established publication [13].

### 4.7. In Vitro Anti-Inflammatory Assays via Mammalian Target of Rapamycin (mTOR) Kinase, AMP-Activated Protein Kinase (AMPK), and Tumor Necrosis Factor-Alpha (TNF-α) Expressions

In vitro evaluation for mTOR, AMPK, and TNF-α refers to the protocol of each kit provided by the manufacturer and is in line with the established and modified study protocol [45]. First, 25 μg/μL of a RAW264.7 cell line murine macrophage (ATCC: American Type Culture Collection, Rockville, MD, USA) was combined with the right amount of 1 SDS sample buffer × (0.5 M Tris-HCl in pH 6.8, 20% SDS, 10% [*v*/*v*] glycerol (C_3_H_8_O_3_), 5% [*v*/*v*] β-mercaptoethanol (HOCH_2_CH_2_SH), and 0.2% bromophenol blue), heated (95 °C) for 5 min, separated by SDS-PAGE, then transferred to a polyvinylidene difluoride membrane. The membrane was blocked with 5% lean, dry milk (*w*/*v*) in a saline-buffered Tris with a Tween buffer (T-TBS) (20 mmol/L Tris-HCl, 0.138 mol/L Sodium chloride (NaCl) (pH 7.6), and 0.1% Tween 20) for the TNF-α, total AMPK, and mTOR detection, with 5% (*w*/*v*) albumin (bovine serum albumin or BSA) in T-TBS for phospo-AMPKα and phospo-mTOR. The expression of TNF-α and mTOR/AMPK activation was analyzed by incubating the membrane in the presence of specific primary antibodies and, subsequently, secondary peroxidase-conjugated antibodies, suitably diluted in 5% BSA in T-TBS. Subsequently, lipopolysaccharide (LPS) from *Pseudomonas aeruginosa* (Sigma-Aldrich, Darmstadt, Germany), prepared in sterile PBS (pH 7.4), was added to the wells at a final concentration of 10 μg/mL, except for the normal control wells (cells without treatment). The cells were then incubated for an additional 24 h before being used in an in vitro anti-inflammatory evaluation trial. More precisely, 5000 cells per well were seeded in the volume of the medium so that the final volume was 100 μL/well. The cells were treated with three green algae extracts (25 μM samples: CrE, CrEA, and CrNH) and incubated for 6 and 24 h, and through analysis, we obtained the percentage values compared to the controls, lipopolysaccharides (cells induced by LPS). Primary antibodies were used overnight at 4 °C under the following conditions: anti-TNF-α, rabbit (1:1000); total-AMPK, rabbit (1:200); antiphospo-mTOR, rabbit (1:1000); antiphospo-AMPKα, rabbit (1:200); total-mTOR, rabbit (1:1000). Secondary antibodies were instead used at two different dilutions: goat anti-rabbit (1:10,000) for TNF-α and total and phospo-mTOR, goat anti-rabbit (1:2000) for total and phospo-AMPK.

### 4.8. Antioxidant Activity by DPPH and ABTS Radical Scavenging Activity Assay

Antioxidant activity in the (2,2-diphenyl-1-picrylhydrazyl radical scavenging activity [DPPH radical, C_18_H_12_N_5_O_6_^+^], BioVision-USA) test was assayed according to the protocol of Permatasari [46]. In the testing vial, concentrations of 50, 100, 150, 200, and 250 μg/mL of CrE, CrEA, and CrNH samples were added to the DPPH reagent (3 mL). The DPPH sample mixtures were then cooled at room temperature for 30 min. Change in the concentration of DPPH was observed based on 517 nm absorbance.

The scavenging of 2,2′-Azino-bis(3-ethylbenzothiazoline-6-sulfonic acid [ABTS+, C_18_H_24_N_6_O_6_S_4_] Sigma-Aldrich, Darmstadt, Germany) or the diammonium salt radical cation was determined based on the procedure by Permatasari [46]. Potassium persulfate (K_2_S_2_O_8_; 2.4 mM) and 7 mM ABTS were mixed at a ratio of 1:1, protected from light with aluminum foil, and allowed to react at 22 °C for 14 h. The mixture was further diluted (e.g., 1 mL of the stock solution plus 60 mL of EtOH [ethanol, C_2_H_6_O]) to obtain a working solution with an absorbance of 0.706 at 734 nm. A fresh working solution was prepared for each test. The (CrE, CrEA, and CrNH) samples were kept in gradients of 50 μg/mL, 100 μg/mL, 150 μg/mL, 200 μg/mL, and 250 μg/mL to be diluted with an ABTS working solution (1 mL), and the absorbance was measured after 7 min at 734 nm. Inhibition of DPPH and ABTS was expressed as a percentage (%) and determined according to the formula below:

(2) $$Inhibition\;Activity\left(\%\right)=\frac{A0-A1}{A0}\times 100\%$$

*A*0 = absorbance of blank; *A*1 = absorbance of standard or sample.

To ensure the validity of the resulting data (ABTS and DPPH), each sample was performed three times (triplicate tests; *n* = 3). Trolox (C_14_H_18_O_4_; PubChem CID: 40634), a known antioxidant molecule, was used as a positive control in ABTS and DPPH. The half-maximal effective concentration ratio (EC_50_) was used to express the radical scavenging capability of CrE, CrEA, and CrNH samples and Trolox, which is defined as the concentration of a sample that causes a 50% decrease in the initial radical concentration. Inhibition of DPPH and ABTS assays was expressed as a percentage and determined according to the formula described in the literature [46].

### 4.9. Management Data and Statistical Analyses

All statistical analyses were carried out using GraphPad Prism 9 Premium Software MacBook version; data are shown as calculated mean and standard deviation (means ± SD). The EC_50_ was analyzed using a GraphPad Premium statistical analysis package “non-linear regression (log[inhibitor] vs. normalized response)–variable slope” to statistically assess data from in vitro experiments, including antioxidant inhibition of DPPH, ABTS, antidiabetic, anti-obesity, and cytotoxicity, and they were all performed three times (thrice). In particular, the evaluation of TNF-α expression and AMPK/mTOR activation was carried out by applying a two-way ANOVA. The value of *p* < 0.05 was considered statistically significant (95% CI).

## 5. Conclusions

Something new has been reported in this work, starting from the composition of carotenoids and the biological activity of *Caulerpa racemosa* in various solvent fractions (CrE: *Caulerpa racemosa*—ethanol; CrEA: *Caulerpa racemosa*—ethyl acetate; CrNH: *Caulerpa racemosa*—*n*-hexane). Of the seven carotenoids observed, it should be highlighted that β-carotene and canthaxanthin were the two most dominant carotenoids present in the *C. racemosa*. Interestingly, the carotenoid extract of *C. racemosa* has good biological activity in inhibiting α-glucosidase (an EC_50_ of CrNH = 52.76 µg/mL), α-amylase (an EC_50_ of CrE = 69.12 µg/mL), lipase (an EC_50_ of CrE = 45.51 µg/mL), DPPH (an EC_50_ of CrE = 52.84 µg/mL) and ABTS (an EC_50_ of CrE = 79.73 µg/mL), and the TNF-α and mTOR, as well as upregulating the AMPK, which makes it a candidate drug or functional antidiabetic food and a very promising anti-obesity and anti-inflammatory. More interestingly, besides having a good efficacy value, the cytotoxicity value of the carotenoid extract of *C. racemosa* shows a level of safety (with a lethal concentration > 500 μg/mL) in normal cells, which makes it a potential for the further development of nutraceuticals and pharmaceuticals. However, further studies are needed to isolate and purify each of the observed carotenoids from the green algae *Caulerpa racemosa*, such as by continuing molecular simulation or in silico studies and in vivo tests, which are being planned by authors to observe the efficacy at advanced levels before being commercialized at industrial levels.

## Figures and Tables

**Table 1:** Measured theoretical and accurate masses of the targeted carotenoids in *C. racemosa* by Orbitrap-MS.

Observed Compounds	RT (min)	Selected Ion	Observed MW	Peak	PubChem CID	Molecular Formula	* CAS Number
Fucoxanthin	16.55	[M + H–H_2_O]^+^	658.8780	1	5281239	C_42_H_58_O_6_	3351-86-8
Lutein	19.20	[M + H–H_2_O]^+^	568.4237	2	5281243	C_40_H_56_O_2_	127-40-2
Astaxanthin	20.15	[M + H]^+^	596.5901	3	5281224	C_40_H_52_O_4_	472-61-7
Canthaxanthin	22.31	[M + H]^+^	564.7006	4	5281227	C_40_H_52_O_2_	514-78-3
Zeaxanthin	21.18	[M]^+^	568.7206	5	5280899	C_40_H_56_O_2_	144-68-3
β-Cryptoxanthin	23.70	[M]^+^	552.8836	6	5281235	C_40_H_56_O	472-70-8

RT: Retention time (minutes); MW: molecular weight; * CAS: Chemical Abstracts Service from the literature. CAS number for β-carotene: 7235-40-7. PubChem CID for β-carotene: 5280489.

**Table 2:** Carotenoids observed in dry weight (mg/100 g) of *C. racemosa* via UHPLC-ESI-MS analysis.

Samples	β-Carotene	β-Cryptoxanthin	Fucoxanthin	Astaxanthin	Canthaxanthin	Zeaxanthin	Lutein
CrE	20.50 ± 0.10 ^x^	2.30 ± 0.10 ^x^	1.40 ± 0.01 ^x^	4.60 ± 0.10 ^x^	15.70 ± 0.50 ^x^	4.70 ± 0.01 ^x^	1.50 ± 0.50 ^x^
CrEA	11.70 ± 0.00 ^y^	4.00 ± 0.50 ^y^	2.70 ± 0.01 ^y^	3.00 ± 0.50 ^y^	9.55 ± 0.05 ^y^	6.50 ± 0.10 ^y^	4.50 ± 0.05 ^y^
CrNH	6.50 ± 0.55 ^z^	7.00 ± 0.01 ^z^	5.50 ± 0.50 ^z^	7.90 ± 0.05 ^z^	3.50 ± 0.01 ^z^	9.00 ± 0.05 ^z^	5.50 ± 0.55 ^y^

CrE: *C. racemosa*—ethanol (polar); CrEA: *C. racemosa*—ethyl acetate (semi-polar); CrNH: *C. racemosa*—*n*-hexane (non-polar); values are presented as means ± SD of triplicate analysis (*n* = 3). Letters (x, y, z) denote significant differences (*p* < 0.05; 95% CI; one-way ANOVA) between the mean values within the same column.

**Table 3:** The half-maximal lethal concentration (LC_50_) value of carotenoid extract of *C. racemosa* on a cytotoxicity test in BUD-8 normal cell lines.

Hours of Incubation	LC_50_ (μg/mL)		
	CrE	CrEA	CrNH
24 h	2314.00	2835.11	1351.29
48 h	1568.00	1916.50	1001.54

CrE: *C. racemosa*—ethanol (polar); CrEA: *C. racemosa*—ethyl acetate (semi-polar); CrNH: *C. racemosa*—*n*-hexane (non-polar).

**Table 4:** Modulation in the expression of mTOR, AMPK, and TNF-α by carotenoid extract of *C. racemosa* compared to the control, lipopolysaccharide.

			6 h			24 h	
	Markers	CrE	CrEA	CrNH	CrE	CrEA	CrNH
% Increase	AMPK	30.96	21.16	25.00	54.20	41.76	38.46
% Decrease	TNF-α	28.73	16.13	15.83	49.70	41.40	39.46
% Decrease	mTOR	25.63	24.53	24.96	41.90	39.93	38.76

% Increase or decrease compared to the control, lipopolysaccharide (LPS); 6 h: six-hour incubation; 24 h: twenty-four-hour incubation. CrE: *C. racemosa*—ethanol (polar); CrEA: *C. racemosa*—ethyl acetate (semi-polar); CrNH: *C. racemosa*—*n*-hexane (non-polar).

## Data Availability

The data sets generated and/or analyzed in this study are available in the manuscript or can be requested from the author (F.N., R.K. and N.A.T.) upon reasonable request.

## Supplementary Materials

The following supporting information can be downloaded at: https://www.mdpi.com/article/10.3390/molecules28073267/s1, Table S1. Molecular structure visualization of observed carotenoids in *Caulerpa racemosa*.

## Sample Availability

Samples of the compounds are not available from the authors.
