# Supplementary material for: Carotenoids Composition of Green Algae Caulerpa racemosa and Their Antidiabetic, Anti-Obesity, Antioxidant, and Anti-Inflammatory Properties
Source: Molecules. 2023 Apr 6;28(7):3267. doi: 10.3390/molecules28073267 (PMC10096636; doi:10.3390/molecules28073267)
Supplement: Supplementary file 1 [file molecules-28-03267-s001.zip › molecules-2314385-supplementary.pdf]

Table S1. Molecular structure visualization of observed carotenoids in *Caulerpa racemosa*

**Compounds**	**2D**	**3D**
β-Carotene	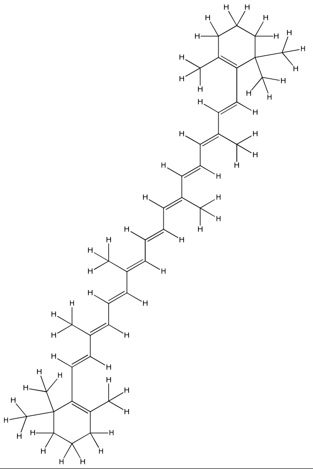	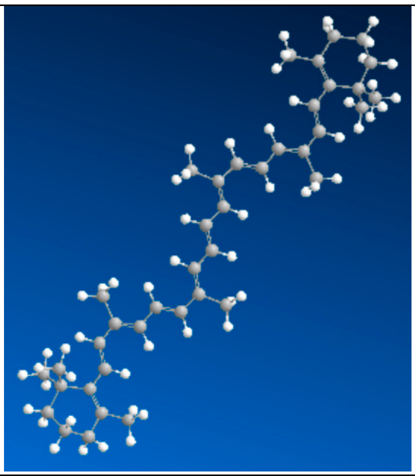
β-Cryptoxanthin	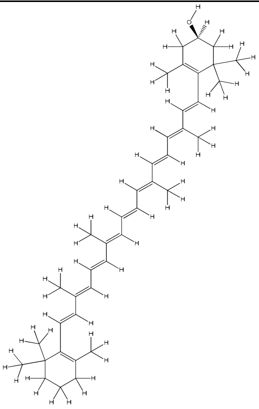	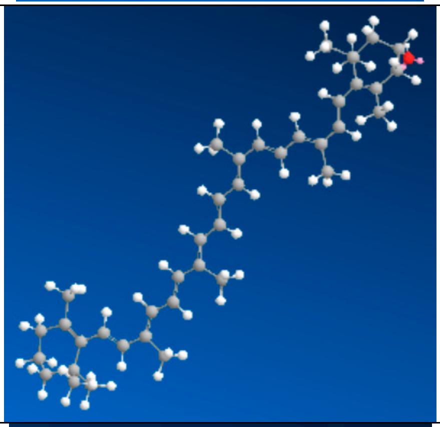
Fucoxanthin	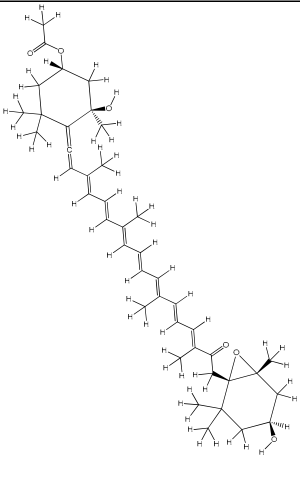	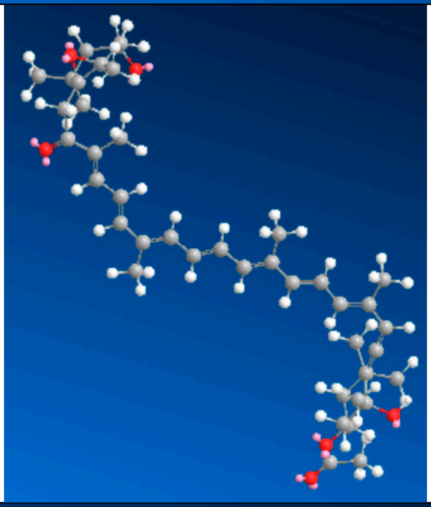
Astaxanthin	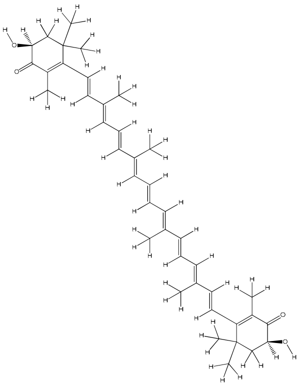	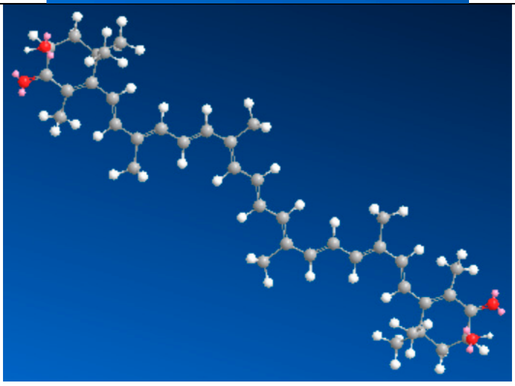

Canthaxanthin	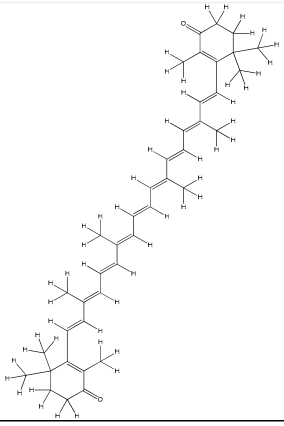	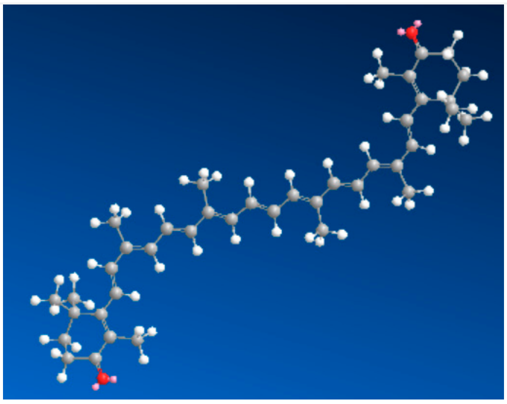
Zeaxanthin	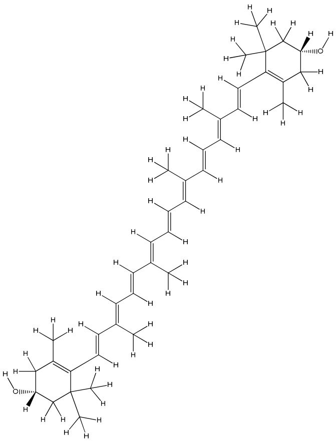	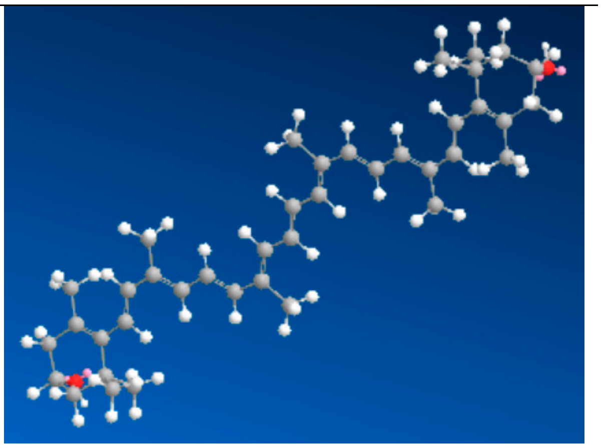
Lutein	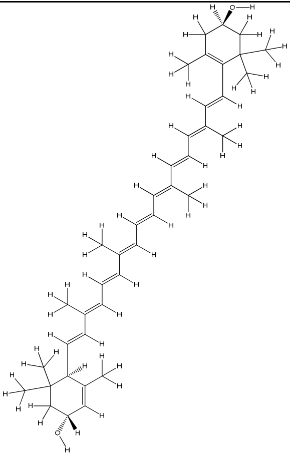	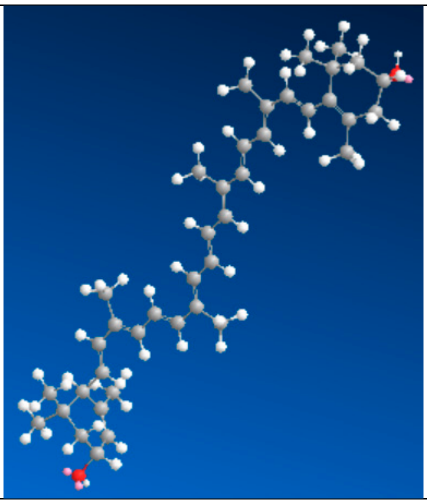
